# Epidemiology of Non-O157 Shiga Toxin-Producing *Escherichia coli* in the Province of Alberta, Canada, from 2018 to 2021

**DOI:** 10.3390/microorganisms10040814

**Published:** 2022-04-14

**Authors:** Heather Glassman, Christina Ferrato, Linda Chui

**Affiliations:** 1 Department of Laboratory Medicine and Pathology, University of Alberta, Edmonton, AB T6G 2R3, Canada; hglassma@ualberta.ca; 2 Alberta Precision Laboratories-Public Health Laboratory (ProvLab), Calgary, AB T2N 4W4, Canada; christina.ferrato@albertaprecisionlabs.ca; 3 Alberta Precision Laboratories-Public Health Laboratory (ProvLab), Edmonton, AB T6G 2J2, Canada

**Keywords:** Shiga toxin-producing *Escherichia coli*, non-O157, virulence factors, epidemiology

## Abstract

Non-O157 serogroups contribute significantly to the burden of disease caused by Shiga toxin-producing *Escherichia coli* (STEC) and have been underrecognized by traditional detection algorithms. We described the epidemiology of non-O157 STEC in Alberta, Canada for the period of 2018 to 2021. All non-O157 STEC isolated from clinical samples were submitted for serotyping and qPCR targeting the *stx*_1_ and *stx*_2_ genes. A total of 729 isolates were identified. Increased detection occurred over the summer months, peaking in July. Patients 18 years and younger made up 42.4% of cases, with 31.1% in those 0–9 years of age. There was a slight female predominance (399/729, 54.7%) A total of 50 different serogroups were detected; the most common were O26 (30.3%), O103 (15.9%), O111 (12.8%), O121 (11.0%), O118 (3.3%) and O71 (2.9%). These six serogroups made up 76.2% of all isolates. In total, 567 (77.8%) were positive for *stx*_1_, 114 (15.6%) were positive for *stx*_2_ and 48 (6.6%) were positive for both *stx*_1_ and *stx*_2_. A wide variety of non-O157 serogroups have been detected in Alberta, with the most frequent serogroups differing from other locations. These results highlight the need for further characterization of their virulence factors and clinical impact.

## 1. Introduction

Shiga toxin-producing *Escherichia coli* (STEC) are a group of pathogenic *Escherichia coli* (*E. coli*) responsible for a spectrum of disease that includes hemorrhagic colitis and hemolytic uremic syndrome (HUS) [1].

STEC can be broadly categorized into O157 and non-O157 serogroups based on their O surface antigen type. A heterogeneous group of over 400 non-O157 serogroups have been recognized, of which more than 100 have been associated with human gastrointestinal disease [2]. Although the clinical relevance of O157:H7 has been well recognized, the significance of non-O157 serogroups was established following a publication by the Centers for Disease Control and Prevention (CDC), which recognized the six most prevalent non-O157 serogroups recovered in their surveillance (O26, O103, O111, O121, O45 and O145) and recommended that clinical laboratories implement stool culture methods for the detection of both O157 and non-O157 STEC serogroups [3].

Classical STEC detection algorithms were primarily targeted at biochemical differences specific to O157 STEC and missed a significant proportion of non-O157 STEC [4]. Newer detection algorithms have been implemented with the aim to improve the detection of non-O157 serogroups [5]. The 2017 Infectious Diseases Society of America (IDSA) guidelines recommended that STEC screening should include an enzyme immunoassay (EIA) or nucleic acid amplification test (NAAT) for the detection of non-O157 along with O157 strains [6]. In 2018, the Canadian Public Health Laboratory Network (CPHLN) published recommendations for the detection of STEC including both O157 and non-O157 [5]. These guidelines recommend that microbiology laboratories utilize either a chromogenic agar or culture-independent test, such as an EIA, to detect the presence of Shiga toxins, or an NAAT targeting the Shiga toxin genes to improve detection of all STEC in clinical samples [5]. These diagnostic methods were adopted for STEC detection at Alberta Precision Laboratories-Provlab (APL-ProvLab) prior to the start of this study [7]. Laboratories submitting to the APL-ProvLab adopted these guidelines during the study period.

The pathogenicity of STEC is conferred by the production of Shiga toxins. These toxins are the major virulence determinants of STEC and act by inhibition of protein synthesis [8]. There are two types of Shiga toxins: Stx1 (comprised of subtypes 1a,c,d) and Stx2 (comprised of subtypes 2a–2g) [9]. The *stx* genes are carried by bacteriophages and their expression is linked to the phage life cycle [8]. Stx2 has been more strongly associated with severe disease and HUS [10]. The Shiga toxin type and subtype produced varies between and within serogroups, so although non-O157 as a group have been associated with less severe disease than O157, the degree of severity and outbreak potential can vary considerably within members of the group [11]. The most prevalent serogroups have been shown to vary by geographic location [12].

In this study, we aimed to analyze all non-O157 STEC isolates recovered from clinical stool samples in Alberta, Canada from 2018 to 2021. We retrospectively reviewed their demographic data, serotypes, and Shiga toxin gene (*stx*) profile to describe the epidemiology of non-O157 STEC in our province.

## 2. Materials and Methods

### 2.1. Submission and Testing of Clinical Samples

Alberta is a province of 4.4 million people located in western Canada. STEC infection is a notifiable disease in Alberta and is reportable to the public health authority [13]. All microbiology laboratories in Alberta detect STEC using a combination of methods according to the CPHLN recommendations published in 2018 [5]. All stools submitted to two major frontline microbiology laboratories were screened by PCR, followed by culture onto CHROMagar™ STEC (Dalynn Biologicals, Calgary, AB, Canada). A sweep of mauve color colonies, indicative of STEC, were inoculated into enrichment broths as specified by the submitting laboratory and incubated overnight at 37 °C. An EIA (SHIGA TOXIN QUIK CHEK^TM,^ TechLab, Blacksburg, VA, USA) of the overnight enriched culture was done to confirm the presence of STEC before submission of the broths to APL-ProvLab for isolation.

Following receipt at APL-ProvLab, 100 μL of the overnight enriched culture was inoculated onto CHROMagar™ STEC (Dalynn Biologicals, Calgary, AB, Canada) agar and MacConkey agar. After 20 to 24 h of incubation at 37 °C, plates were examined for the presence of mauve color colonies which are indicative of STEC. Between 3 and 5 colonies were selected and subjected to individual colony PCR for detection of the Shiga toxin genes (*stx*_1_ and *stx*_2_). Each colony was suspended in 100 μL of rapid lysis buffer (100 mM NaCl, 10 mM Tris-HCL pH 8.3, 1 mM EDTA pH 9.0, 1% Triton X-100) and heated to 95 °C for 15 min using a heating block. The suspension was centrifuged at 13,000× *g* for 10 min and 5 μL of the supernatant was used as a template for qPCR. The primer and probe (Integrated DNA Technology, Skokie, IL, USA) sequences used for amplifying *stx*_1_ and *stx*_2_ are shown in Table 1. The total reaction contained 10 μL of 2X PrimeTime^®^ Gene Expression Master Mix (Integrated DNA Technology, Skokie, IL, USA), 0.33 μM of each primer, 0.22 μM probe, 5μL DNA template and molecular biology grade water in a total of 20 μL reaction volume. Positive DNA for *stx*_1_+/*stx*_2_+ and no template controls were included in each run. The qPCR amplification conditions consisted of 95 °C for 3 min, followed by 40 cycles of 95 °C for 5 s and 60 °C for 30 s, performed on the 7500 FAST real-time PCR system (Applied Biosystems, Foster City, CA, USA). One selected PCR positive colony per sample was submitted to the Public Health Agency of Canada-National Microbiology Laboratory (PHAC-NML) in Winnipeg, Manitoba, Canada for conventional serotyping by agglutination.

### 2.2. Data Collection

All non-O157 STEC strains isolated at APL-ProvLab were prospectively catalogued along with their test results in the laboratory secured database. Demographic data were captured from the requisition at the time of stool specimen submission. No clinical information on disease severity or outcomes was available.

### 2.3. Inclusion and Exclusion Criteria

All non-O157 STEC isolated from clinical cultures from January 2018 until December 2021 were included. Repeat isolations from the same patient received within one month of the initial sample were excluded.

## 3. Results

A total of 729 non-O157 STEC isolates were reported within the 4-year period from January 2018 until the end of December 2021. The annual incidence rate per 100,000 population was between 3.5 and 3.7 for 2018, 2020 and 2021. However, the incidence rate for 2019 was higher at 5.8 per 100,000.

### 3.1. Serogroups and Serotypes Identified

A total of 50 different serogroups were identified (Supplementary Table S1). The most prevalent were O26 (221/729, 30.3%), O103 (116/729, 15.9%), O111 (93/729, 12.8%), O121 (80/729, 11.0%), O118 (24/729, 3.3%) and O71 (21/729, 2.9%). Other serogroups made up 22.9% of the cases (167/729). In the case of 7/729 isolates (0.9%) the serogroup was unknown as they were unresolved by conventional serotyping.

The distribution of serogroups and serotypes is summarized in Table 2. The majority of O26 identified as O26:H11 (155/221, 70.1%), followed by O26:H non-motile (65/221, 29.4%); 1 was O26:H undetermined. For O103, the majority were O103:H2 (80/116, 69.0%) and O103:H25 (20/116, 17.2%), with the remainder split between O103:H11 (8/116, 6.9%), O103:H non-motile, 6/116, 5.2%) and O103:H19 (2/116, 1.7%). All of the O111 typed as O111:H non-motile (93/93, 100%). The O121 were primarily O121:H19 (77/80, 96.3%) with only a few O121:H non-motile (3/80, 3.7%). O118 was split between O118:H16 (10/24, 41.6%), O118:H2 (9/24, 37.5%), O118:H undetermined (3/24, 12.5%), O118:H14 (1/24, 4.2%) and O118:H non-motile (1/24, 4.2%). The majority of O71 were O71:H11 (16/21, 76.2%); the remainder were O71:H8 (3/21, 14.3%) and O71:H non-motile (2/21, 9.5%).

### 3.2. Seasonality

**Figure 1:**
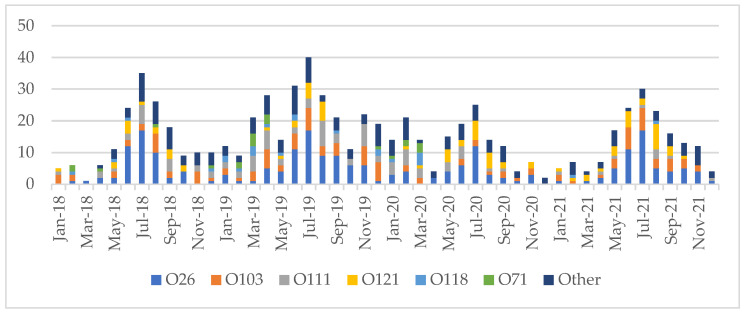
Number of non-O157 isolates detected per month by serogroup.

Non-O157 STEC infection was seen year-round. Incidence was highest from May to September in 2018 (114/159, 71.7%), 2019 (134/254, 52.8%), 2020 (97/155, 62.6%), 2021 (109/161, 67.7%) and overall (454/729, 62.3%). Peak incidence was in July (130/729, 17.8%). The remaining infections were distributed from October to April (range: 35–52/729, 4.8–7.1%). (Figure 1).

### 3.3. Geographic Distribution

**Figure 2:**
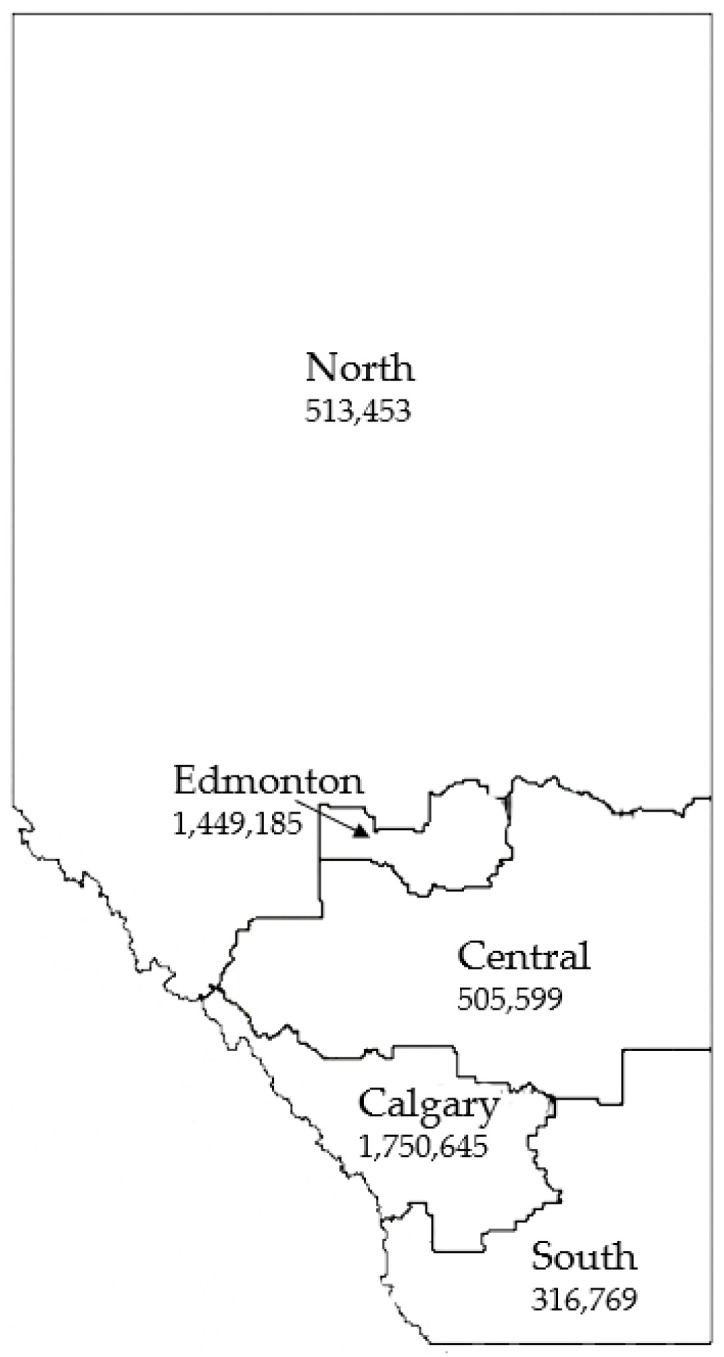
Map of the five health zones in the province of Alberta, Canada with 2021 projected population estimates. [15,16]. Available online: https://geodiscover.alberta.ca/geoportal/#searchPanel (accessed on 26 February 2022) 2022 Government of Alberta.

The majority of non-O157 STEC infections were concentrated in the health authority zones corresponding to the largest urban centers in the province: Calgary (344/729, 47.2%) and Edmonton (171/729, 23.5%). Most of the remainder of the cases were distributed amongst the other zones: North (48/729, 6.6%), Central (77/729, 10.6%) and South (71/729, 9.7%). The rest (18/729, 2.5%) were of out-of-province origin. (Figure 2). 

### 3.4. Age and Sex Distribution

**Figure 3:**
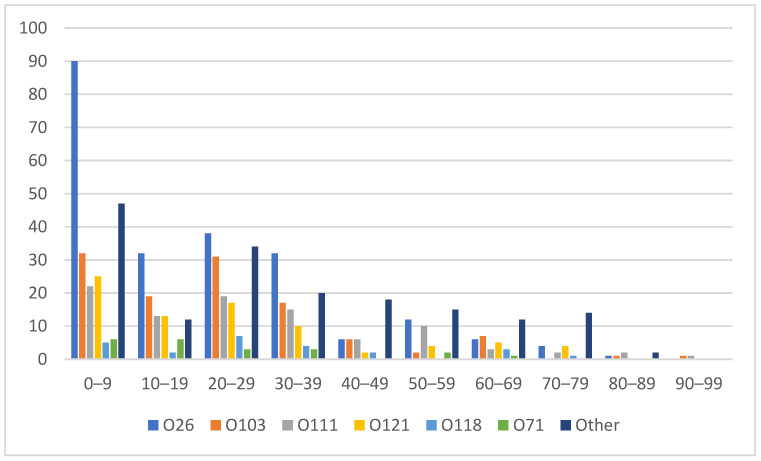
Number of non-O157 cases by serogroup and age.

Patients 18 years of age and younger accounted for 42.4% of all the non-O157 STEC isolates (309/729), with the highest proportion in the 0–9 year age range (227/729, 31.1%). Within the 0–9 year age range, O26 consisted of 40.7% (90/221), followed by O121 (25/80, 31.3%), O71 (6/21, 28.6%), O103 (32/116, 27.6%), O111 (22/93, 23.7%) and O118 (5/24, 20.8%). Other serogroups made up 27.0% (47/174) of the 0–9 year age range. (Figure 3). There was a slight female predominance overall (399/729, 54.7%).

### 3.5. Shiga Toxin Profile

**Figure 4:**
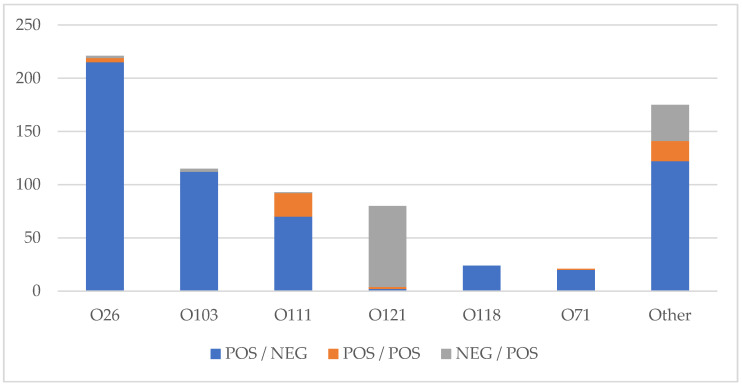
Shiga toxin profiles of STEC isolates by PCR positivity (POS) or negativity (NEG) (*stx*_1_/*stx*_2_).

The majority of isolates, 567/729 (77.8%), were positive for *stx*_1_ and negative for *stx*_2_, 114 (15.6%) were negative for *stx*_1_ and positive for *stx*_2_, and 48 (6.6%) were positive for both *stx*_1_ and *stx*_2_. The *stx*_1_ positive, *stx*_2_ negative profiles were the predominant Shiga toxin profiles seen for O26 (215/221, 97.3%), O103 (113/116, 97.4%) and O118 (24/24, 100%). O111 had a mix of *stx*_1_ positive, *stx*_2_ negative (70/93, 75.2%) and *stx*_1_ positive, *stx*_2_ positive (22/93, 23.7%), with one *stx*_1_ negative, *stx*_2_ positive. O121 was primarily *stx*_1_ negative, *stx*_2_ positive (76/80, 95.0%). (Figure 4)

## 4. Discussion

Non-O157 STEC are gastrointestinal pathogens that confer risk both at the individual and population level due to their potential for severe clinical outcomes such as HUS and their ability to cause outbreaks [2]. Recent recognition of their clinical relevance and the burden of resulting disease has led to changes in stool pathogen detection methods. We have reviewed the epidemiology of non-O157 STEC isolates collected over a four-year period from 2018 to 2021 in the province of Alberta, Canada.

The most prevalent serogroups isolated were O26, O103, O111, O121, O118 and O71. The most common serogroups reported in the literature varies by geographic origin. The most prevalent serogroups seen in the CDC FoodNet data from 2000 to 2010 were O26, O103, O111, O121, O45 and O145 [3]. An update of their data from 2004 to 2014 showed the same serogroups in the same order [12]. Data from England demonstrated O26, O146, O91, O128, O103 and O117 as their most prevalent serogroups [2]. A European surveillance report in 2019 stated serogroup O26 was the most common, followed by O146, O103 and O91 [17]. A study from Switzerland recorded that the top four serogroups in their country were O103, O26, O91 and O80 [18]. Data from Korea showed O103, O26, O91 and O8 [19] and Japan reported O26, O111, O103, O121 and O145 [20] as their predominant serogroups. British Columbia, Canada (located west of Alberta) found their top serogroups to be O121, O26, O103, O117 and O111 [21]. Internationally, and within Canada, there is considerable variation in the most common serogroups. Despite this variation, it appears that O26 and O103 may be some of the most prevalent non-O157 serogroups globally.

O157:H7 STEC is the most common serotype in many locations; however, the non-O157 group often outnumbers O157 in clinical specimens when considered in their entirety [22]. This, coupled with the observed decline in O157 incidence in many locations, is demonstrative of the significant clinical burden of non-O157 and the importance of implementing sensitive methods for their detection [21,23,24]. Our study captured 729 cases of non-O157 STEC over a period of 4 years. During the same period, only 492 cases of O157 STEC were detected. In contrast, a study of O157 STEC isolates from the same region during a 5-year period from 2009 to 2016 discovered 801 cases [25]. This is indicative of the large clinical burden of non-O157 infections in Alberta as well as the decline in O157 cases. This is consistent with data from Manitoba, Canada where O157 strains were shown to make up only 28.6% of all STEC isolates [26].

Incidence rates in the present study (3.5–5.8 per 100,000) are significantly higher than those reported in the CDC FoodNet study (increasing over time from 0.12 per 100,000 in 2000 to 0.95 per 100,000 in 2010) [3]. A recent systematic review describes the steadily increasing rates of non-O157 detection, which may reflect the expanded and improved detection methods [12]. Data from 2011 to 2017 in the province of British Columbia, Canada, showed an incidence rate of 3.0 per 100,000, although this included both O157 and non-O157 cases together [21]. Previous studies have shown substantial recovery of O157 and non-O157 STEC from cattle in the USA and Canada [27]. Alberta’s high level of cattle production has been previously considered to contribute to the increased incidence rates of O157 STEC [25]. Prevalence studies have shown that up to 74.5% of cattle hides are positive for non-O157 STEC, including several of the most common serogroups seen in our study, such as O26, O103, O111 and O121 [28]. Overall, the elevated incidence rate of non-O157 STEC seen in this study may represent geographic differences, variation in detection methods/algorithms or a combination of multiple factors. Future study may help to further elucidate these differences.

Our data demonstrated that non-O157 STEC was isolated year-round with increased numbers of cases in the summer months, in keeping with the previous literature [29]. Cases were spread throughout the entire province and were highest in the two main urban centers. This mirrored the pattern observed in a recent analysis of O157 STEC in Alberta [25]. Pediatric patients, particularly those in the 0–9 year age range, made up a high proportion of non-O157 cases. Similar age and sex distributions to our data were described in a recent analysis of data from British Columbia, Canada [21].

The majority of non-O157 STEC cases are known to follow sporadic transmission which has been most strongly associated with undercooked meats and contact with animals or their environments [30]. However, outbreaks such as the 2011 O104 outbreak in Germany have been associated with large numbers of severe clinical disease resulting in hospitalizations, HUS and death [31]. More recently, a 2019 multistate American outbreak of 209 cases of O103 STEC led to 29 hospitalizations and two cases of hemolytic uremic syndrome [32]. In a review of EHEC outbreaks in Japanese childcare facilities from 2010 to 2013, O26 associated outbreaks outnumbered those caused by O157 (29/66 vs. 22/66) [33]. These examples demonstrate the outbreak potential of serogroups that are commonly found locally; however, a synthesis of additional data is required to determine the extent of the risk of individual strains.

The previous literature has demonstrated an increased incidence of severe disease and HUS with *stx*_2_ in comparison to *stx*_1_ [34]. Our data demonstrates a predominance of *stx*_1_ positivity overall, which might be expected to be associated with milder disease. However, O121, which made up 11% of the non-O157 seen in this study, was primarily *stx*_2_ positive. A variety of virulence factors have been found to be involved including toxin subtypes, locus of enterocyte effacement (LEE) and others [10]. This data is not available in the present study but is the subject of further study.

Limitations of this work include the lack of information on clinical severity or outcome. Many of these isolates likely represent clinically relevant disease, resulting in stool sample collection and submission. Notably, available data would suggest that asymptomatic carriage rates of STEC are low [35]. Overall, these numbers are likely to underrepresent the true burden of disease as many cases may not have presented for medical care or had specimens collected for culture.

This work demonstrates the high frequency of non-O157 STEC isolates in our setting, highlighting the importance of algorithms capable of detecting these pathogens. The heterogeneity of this group indicates that further study is required to further assess the virulence factors present in these isolates for a more complete picture of their disease risk. Next steps include analysis of whole genome data for in silico detection of Shiga toxin subtypes and the presence of other known virulence factors for further characterization of these isolates.

## Figures and Tables

**Table 1:** Primer and probe sequences used in this study. Adapted with permission from Perelle et al. (2004) [14]. 2004 Elsevier.

Reference Gene, Primer/Probe	Sequence 5′-3′
*stx*_1_, *stx*_2_-F	TTT GTY ACT GTS ACA GCW GAA GCY TTA CG
*stx*_1_, *stx*_2_-R	CCC CAG TTC ARW GTR AGR TCM ACR TC
*stx*_1_-P	CTG GAT GAT CTC AGT GGG CGT TCT TAT GTA A
*stx*_2_-P	TCG TCA GGC ACT GTC TGA AAC TGC TCC

In the sequences: Y is (C, T), S is (C, G), W is (A, T), R is (A, G), M is (A, C).

**Table 2:** Number of serogroups and serotypes isolated during the study period.

Serogroup	Number of Isolates (%)	Serotype	Number of Isolates (% of Serogroup)
O26	221 (30.3)	H11	155 (70.1)
		H Non-motile	65 (29.4)
		H Undetermined	1 (0.5)
O103	116 (15.9)	H2	80 (69.0)
		H25	20 (17.2)
		H11	8 (6.9)
		H Non-motile	6 (5.2)
		H19	2 (1.7)
O111	93 (12.8)	H Non-motile	93 (100)
O121	80 (11.0)	H19	77 (96.3)
		H Non-motile	3 (3.7)
O118	24 (3.3)	H16	10 (41.6)
		H2	9 (37.5)
		H Undetermined	3 (12.5)
		H14	1 (4.2)
		H Non-motile	1 (4.2)
O71	21 (2.9)	H11	16 (76.2)
		H8	3 (14.3)
		H Non-motile	2 (9.5)
Other	167 (22.9)	-	-
Unknown *	7 (0.9)	-	-
Total	729 (100)	-	-

* Unresolved by conventional serotyping.

## Supplementary Materials

The following supporting information can be downloaded at: https://www.mdpi.com/article/10.3390/microorganisms10040814/s1, Table S1: Full List of non-O157 O Types.
