# Supplementary material for: Epidemiology of Non-O157 Shiga Toxin-Producing Escherichia coli in the Province of Alberta, Canada, from 2018 to 2021
Source: Microorganisms. 2022 Apr 14;10(4):814. doi: 10.3390/microorganisms10040814 (PMC9026152; doi:10.3390/microorganisms10040814)
Supplement: Supplementary file 1 [file microorganisms-10-00814-s001.zip › microorganisms-1662435-supplementary.pdf]

Supplementary Table S1. Full List of non-O157 STEC serogroups isolated during the study period

Serogroup	Number of Isolates	Serogroup	Number of Isolates
O26	221	O8	1
O103	116	O11	1
O111	93	O16	1
O121	80	O19	1
O118	24	O27	1
O71	21	O28	1
O5	20	O41	1
O Undetermined	19	O48	1
O145	16	O59	1
O69	15	O77	1
O186	14	O80	1
O Rough	13	O86	1
O84	12	O100	1
Unknown	7	O108	1
O146	5	O115	1
O85	4	O119	1
O91	3	O128	1
O175	3	O136	1
O177	3	O142	1
O38	2	O156	1
O98	2	O170	1
O109	2	O172	1
O113	2	O174	1
O168	2	O178	1
O182	2	O181	1
O1	1	O183	1
O6	1		
Column Total	703	Column Total	26
**Total**	**729**		
